# Monitoring Open Science as transformative change: Towards a systemic framework

**DOI:** 10.12688/f1000research.148290.1

**Published:** 2024-04-23

**Authors:** Ismael Rafols, Ingeborg Meijer, Jordi Molas-Gallart

**Affiliations:** 1 Centre for Science and Technology Studies, Leiden University, Leiden, The Netherlands; 2 INGENIO (CSIC-UPV), Universitat Politecnica de Valencia, Valencia, Spain

**Keywords:** Open Science, Monitoring, Evaluation, Open Access, Open Data

## Abstract

Following a flurry of policies for Open Science (OS), there is now a wave of initiatives to monitor its adoption. However, the great diversity of understandings and activities related to Open Science makes monitoring very challenging. There is a danger that by focusing on what can be readily observed (e.g. publications) many other OS activities are overlooked (e.g. participation), with a potential narrowing of OS scope, streetlight effects, and deviation from the values of OS. Since Open Science can be understood as a systemic transformation of the research system, we have borrowed concepts from Transformative Innovation Policies frameworks which aim at evaluating socio-technical transitions. In accordance with this view of OS as a systemic transformation, we propose that the new monitoring efforts should shift towards: (i) **systemic perspectives** which considers the various actions related to OS, including policies and outputs (e.g. datasets) but also processes (e.g. participatory events), outcomes (e.g. citizen interest in science) and expected impacts (e.g. better scientific contributions to addressing societal problems); (ii) implementation of monitoring as reflexive **learning** (rather than accountability or benchmarking); (iii) mapping the **directionality of the activities and the values associated** with the choices in directions. In summary, a monitoring framework for OS requires a profound change in conventional monitoring practices. The scope should broaden from current focus on outputs (such as publications) towards the processes of connection that make science ‘open’ (usage, co-creation and dialogue), as well as towards outcomes (changes in practices) and the longer-term impacts that reflect the values and normative commitments of OS.

## 1. Introduction

In the wake of a flurry of policies and investments in Open Science (OS), there is now a wave of efforts to monitor its promotion and adoption. Institutions such as the European Commission ^1^, the French ^2^ and Finish ^3^ governments, and the European Open Science Cloud (EOSC) ^4^ are gathering statistics on OS outputs, policies and practices and making them available in dedicated websites. The European Commission has funded a string of projects aimed at capturing and understanding uptake and effects of Open Science, for example through the development of indicators for research assessment ( Opus and GraspOS projects) and on the impacts of OS ( PathOS project). The UNESCO has a Working Group to monitor the implementation of its 2021 Recommendations on OS ( UNESCO, 2021, p. 33) for the 193 countries that signed it ^5^ and has published its first outlook of OS activities in December 2023 ( UNESCO, 2023).

Monitoring Open Science poses major challenges. Different stakeholders hold disparate understandings of OS, often associated with different conceptual models and interests about what science is and aims to accomplish. Most national and institutional policies on OS are recent and thus changes are incipient, with a degree of engagement that varies largely across geographies, disciplines and topics. Some developments are hotly contested, since they run against some of the principles of equity and inclusion espoused by OS, such as open access of publication via Article Processing Charges (APCs) ( Ross-Hellauer et al., 2022). While the dimensions of open access (OS) publications and open data (OD) sets are covered, many other relevant activities, such as engagement with societal actors, are seldom covered and others, like open educational resources, are just beginning to be scanned in a few pioneering countries. There is the danger that this lack of monitoring attention has a ‘street-light effect’ against the adoption of these activities ( UNESCO, 2023).

Yet in our view, the main challenge is that ‘monitoring OS’ also requires a transformation of what ‘monitoring science’ means, since the shift towards OS implies a deep transformation of science itself. In this paper, we propose that this change in the understanding of monitoring cannot be a replacement of old output indicators with new output indicators, as it is often assumed. Instead, new conceptual frameworks and practices for monitoring are required that align with the conceptual shifts associated with OS. We suggest that conceptual frameworks from recent work on evaluation of Transformative Innovation Policies can be useful for thinking how to monitor OS ( Haddad & Bergek, 2023; Molas-Gallart et al., 2021). These frameworks propose to shift efforts towards understanding monitoring as learning, as awareness of values and directionality, with more focus on processes and outcomes.

This study is prompted by our engagement with the UNESCO Working Group on Monitoring OS ( UNESCO, 2023). Therefore, it aims to gain insights on the overall progress of OS across the world, which results from a large variety of national and institutional policies and social developments, rather than assessing specific initiatives. Given that UNESCO’s OS framework is broader and more explicitly normative than many previous OS policies, we bring to the fore the need to monitor OS of transformative change at a global scale.

A number of OS monitors are already in place. The most relevant feature is that they tend to focus on the outputs of research (publications, data, software, etc.) that are accessible from digital platforms. While this is important, we propose that monitors should embrace an understanding of OS as a transformation of the research systems, and therefore encompass a broader range of dimensions, which include:

• **policies** for OS.
• **OS outputs** such as publications and datasets,
• **OS processes** such as increasing collaborative practices (including public engagement) and evaluation approaches in line with OS,
• **OS outcomes** such as increased attention by researchers to societal problems, or evaluations that don’t discriminate by gender or social group,
• **impacts** such as the values highlighted in the list of values of UNESCO OS Recommendation: integrity, inclusion, epistemic diversity, or identifiable contributions for addressing of societal problems.

We first discuss the diversity of activities and the plural (and sometimes conflicting) understandings of OS. Second, we make the case that OS is a systemic transformation of research. Third, we review how models of science are associated with particular monitoring frameworks. Fourth, we describe concepts that can be useful for monitoring OS as a transformation. In particular, we propose:

• to **broaden out the monitoring OS** from a focus on scientific outputs (e.g. datasets), to processes (e.g. participatory events), outcomes (e.g. *better* public engagement) and impacts (e.g. contributions to the achievement of SDGs);
• to adopt **a learning approach to monitoring**, aimed at informing future-oriented strategies, i.e. helping navigate and make choices among options within OS;
• **to consider directionality,** i.e. to open up the monitoring so as to map the different trajectories within each OS dimension (e.g. different colours of the routes to OS), as well as their potential effects and associated values.

In the conclusions we discuss the implications for this perspective for current OS policies.

## 2. What is Open Science? A plurality of views on a diversity of activities

**Figure 1.**
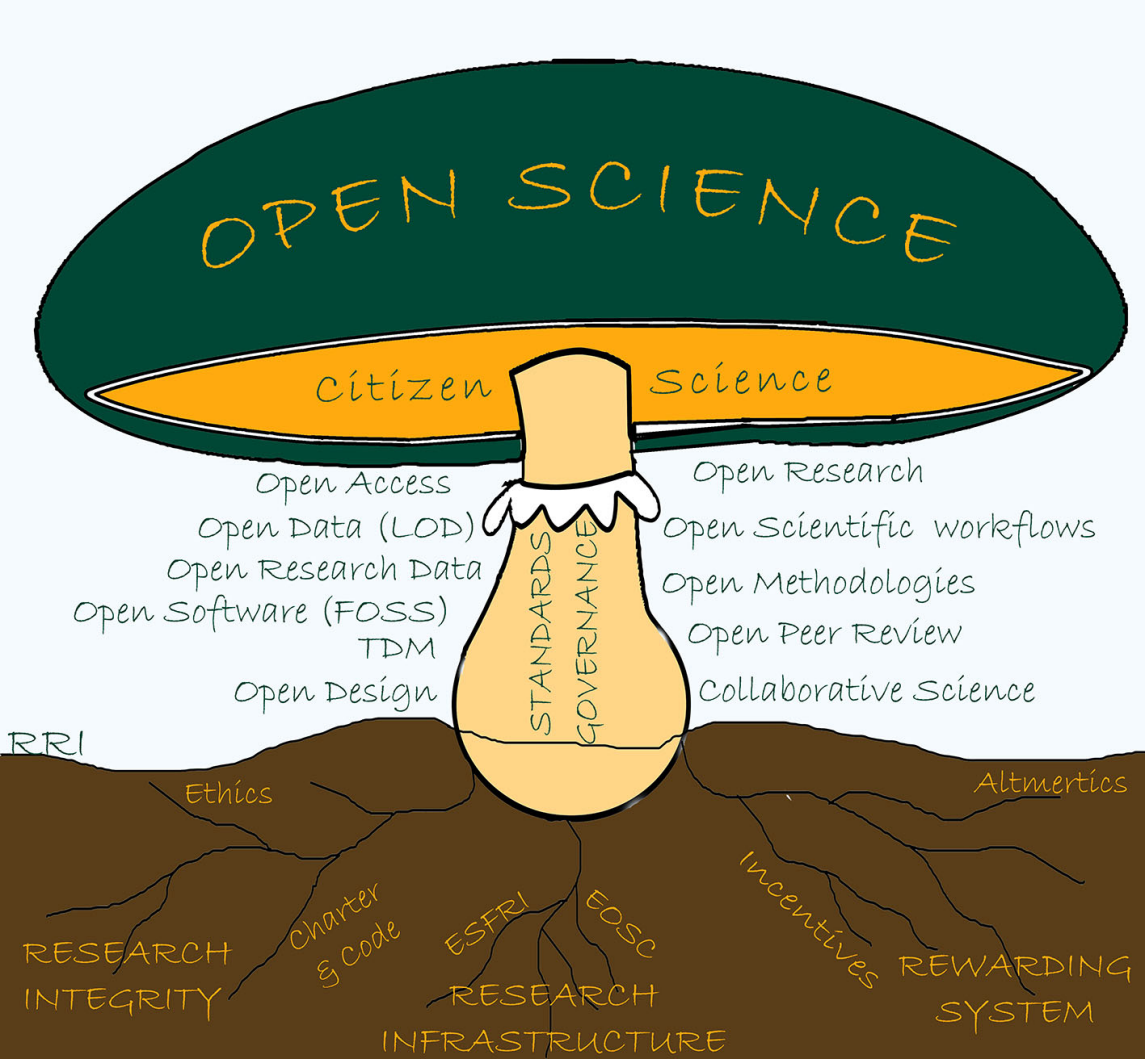
Open Science mushroom, illustrating the diversity of activities and issues involved. Legend: This figure was drawn by Judit Eva’s Fazekas-Paragh based on Eva Méndez (2021, p. 5). Source: https://www.openaire.eu/blogs/hungary-on-the-move-1.

A first difficulty in monitoring Open Science is the vast diversity of activities that it encompasses, from Open Access (OA) publishing to transparency and citizen engagement ^6^. It is often said that OS is an umbrella term or better, a mushroom (as illustrated in Figure 1), in the sense that it consists of many *visible* practical activities (often digital) and that it also concerns, below *its surface,* many underlying institutional issues such as integrity, infrastructure and evaluation.

The programmatic definition of the European Commission (2016, p. 33), highlighted two aspects: first, the mainstream focus on (digital) access and collaborative activities and, second, the expectation that it would transform relations between science and society: ‘Open Science represents a new approach to the scientific process based on cooperative work and new ways of diffusing knowledge by using digital technologies and new collaborative tools. (…) allowing end users to be producers of ideas, relations and services and in doing so enabling new working models, new social relationships and leading to a new modus operandi for science.’ (see ( Leonelli, 2023, p. 18). A definition of OS with these two sides is often found: on the one hand sharing or collaborative (digital) practices; on the other hand an explicit reference to the societal benefits in terms of participation and inclusion ( Arza & Fressoli, 2017).

**Figure 2.**
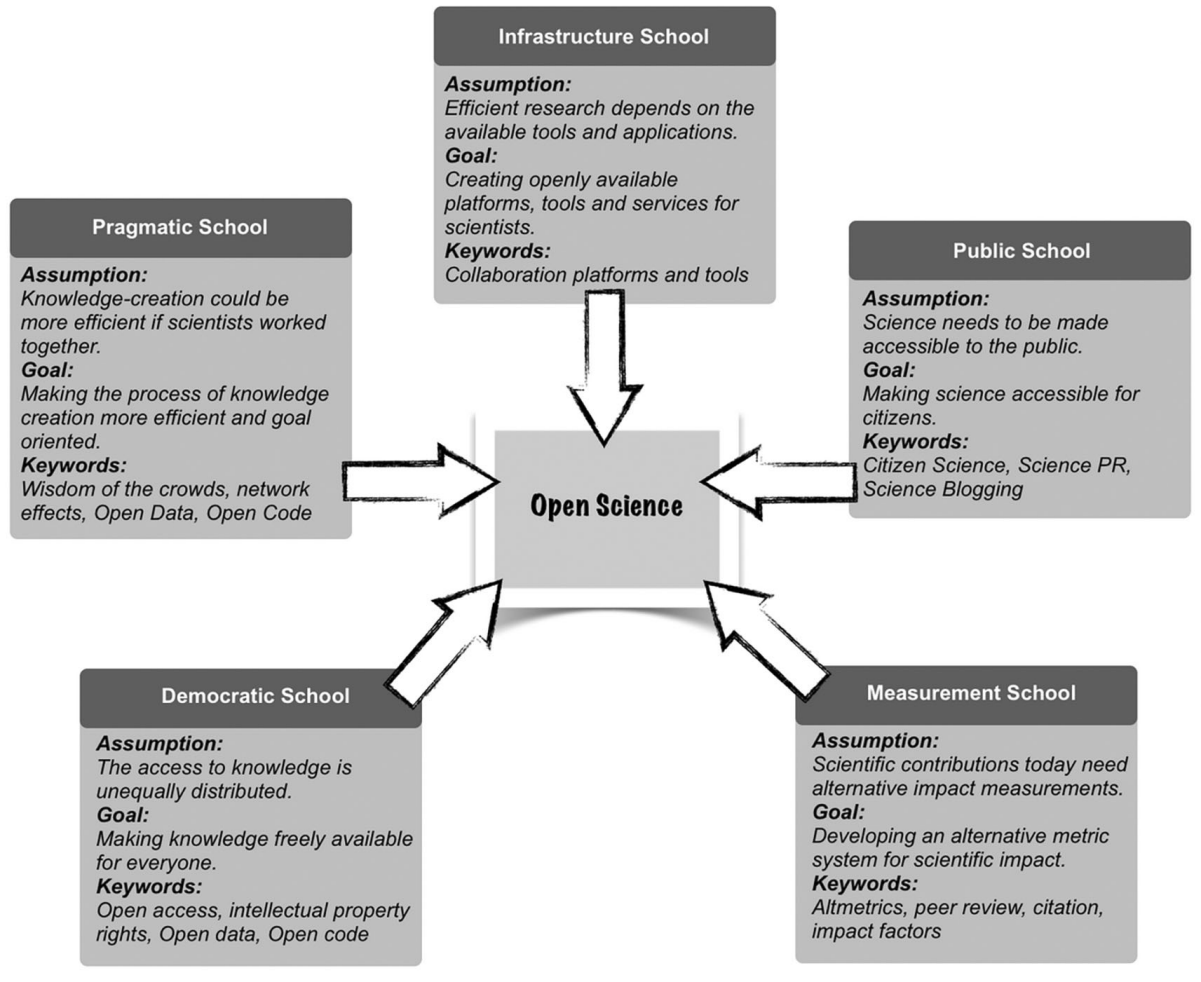
Five schools of thought on Open Science, according to ( Fecher & Friesike, 2014).

Normative commitments to equity and inclusion have been common in the OS movement as launched in 2001 in the Budapest Open Science Initiative ( Ross-Hellauer et al., 2022, p. 3). Nevertheless, definitions and policies have been diverse and ambiguous, depending on the underlying political programmes. For example, whereas many organisations in Latin America highlighted the role of OS for advancing science as a public good ( Babini, 2019), policy adoption in the European context initially viewed the benefits of OS in terms of improvement in quality, efficiency, optimization, integration and potential of science ( Shelley-Egan et al., 2020, p. 11), in a way that emphasized ‘market principles of competition foregrounding its economic role in training the workforce and fostering new products and services’ ( Ross-Hellauer et al., 2022, p. 18). To put some light in this diversity, Fecher and Friesike (2014) proposed five main ‘schools of thought’ (narratives) depending on the focus and goals of OS: infrastructure (platforms), pragmatic (efficiency), public (access), measurement (evaluation) and democratic (free knowledge) schools, as shown in Figure 2. ^7^

There is a strong contrast between the initial aspirations towards global equity of the OS discourse, and the narrower policies on access. Ironically, these policies have actually resulted in greater inequalities in some dimensions, in particular in terms of OA and APCs. This dissonance has led some OS advocates to state that, for all the claimed benefits, the current OS model has not made science more inclusive. Instead, it is still keeping many scientists underrepresented; new technologies exclude those with limited digital rights; and citizens seldom can shape research agendas (see review by Ross-Hellauer et al., 2022). The Manifesto of the Open and Collaborative Network (OCSDNet) ( Albornoz et al., 2020) is an example of this perspective.

In recent years, one can observe policy trends towards a broadening of the OS agenda from more technical aspects of accessibility (e.g. OA and OD) towards more process-oriented aspects (e.g. citizen science and evaluation) and values (diversity and inclusion, the distribution of research benefits). The International Science Council (ISC) has now defined OS as ‘best characterised as the necessary transformation of scientific practice to adapt to the changes, challenges and opportunities of the 21st century digital era to advance knowledge and to improve our world. (…) One of the purposes of Open Science viewed as a call for transformation, is to ensure that ‘no-one is left behind” ( ISC, 2020, p. 2). Also the European Commission states in its Open Science Portal that ‘when partners from across academia, industry, public authorities and citizen groups are invited to participate in the research and innovation process, creativity and trust in science increases.’ ^8^

Some critical voices stress this shift from an understanding of OS as the ‘possibility of access’ towards an understanding of OS as the ‘process of connection’ in accordance with values related to integrity and epistemic justice. Chan proposes that ‘the ability to participate, to connect, and to co-produce knowledge with others who share common concerns is far more important than simply access to content or resources’ ( Chan et al., 2020, p. 2). Leonelli argues that ‘… from being solely a question of sharing resources, openness is thereby conceptualized as the opportunity to make and maintain connections among relevant stakeholders (…) in ways that help to develop ever more relevant forms of interaction with the world’ ( Leonelli, 2023, p. 66). Ross-Hellauer has stressed that enabling access is not enough to foster equity in science in the face of huge disparities in resources ( Ross-Hellauer et al., 2022).

**Figure 3.**
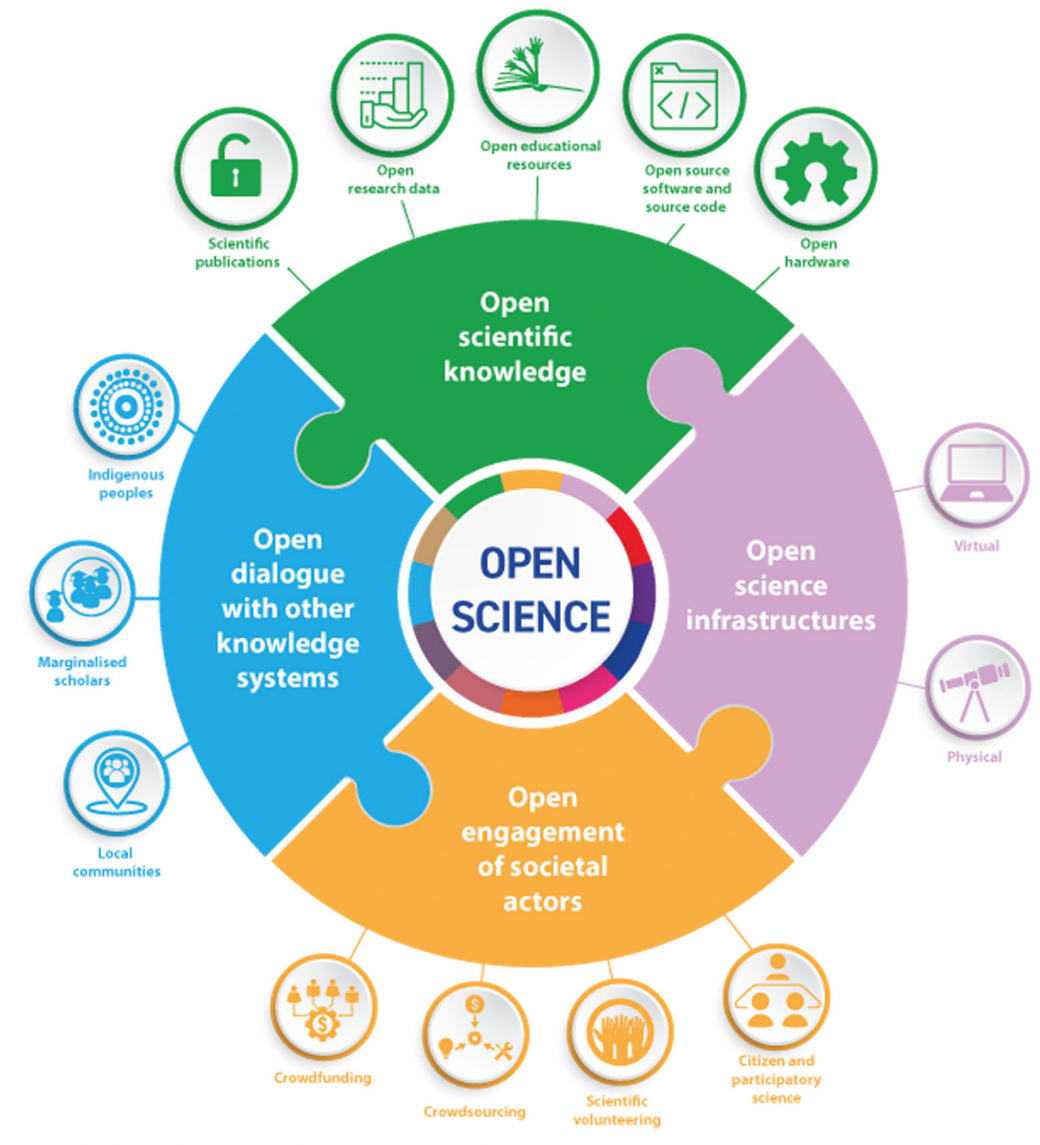
Illustration of the areas of concern mobilised by the UNESCO Recommendation on Open Science. OS is seen as having four pillars: 1) Scientific Knowledge (publications, data, educational resources, software, hardware); 2) Infrastructure (virtual and physical); 3) Engagement of societal actors (citizen and participatory science, volunteering, crowdsourcing, crowdfunding); and 4) Dialogue with other knowledge systems (indigenous peoples, marginalised scholars and local communities). Source: UNESCO (2021).

**Figure 4.**
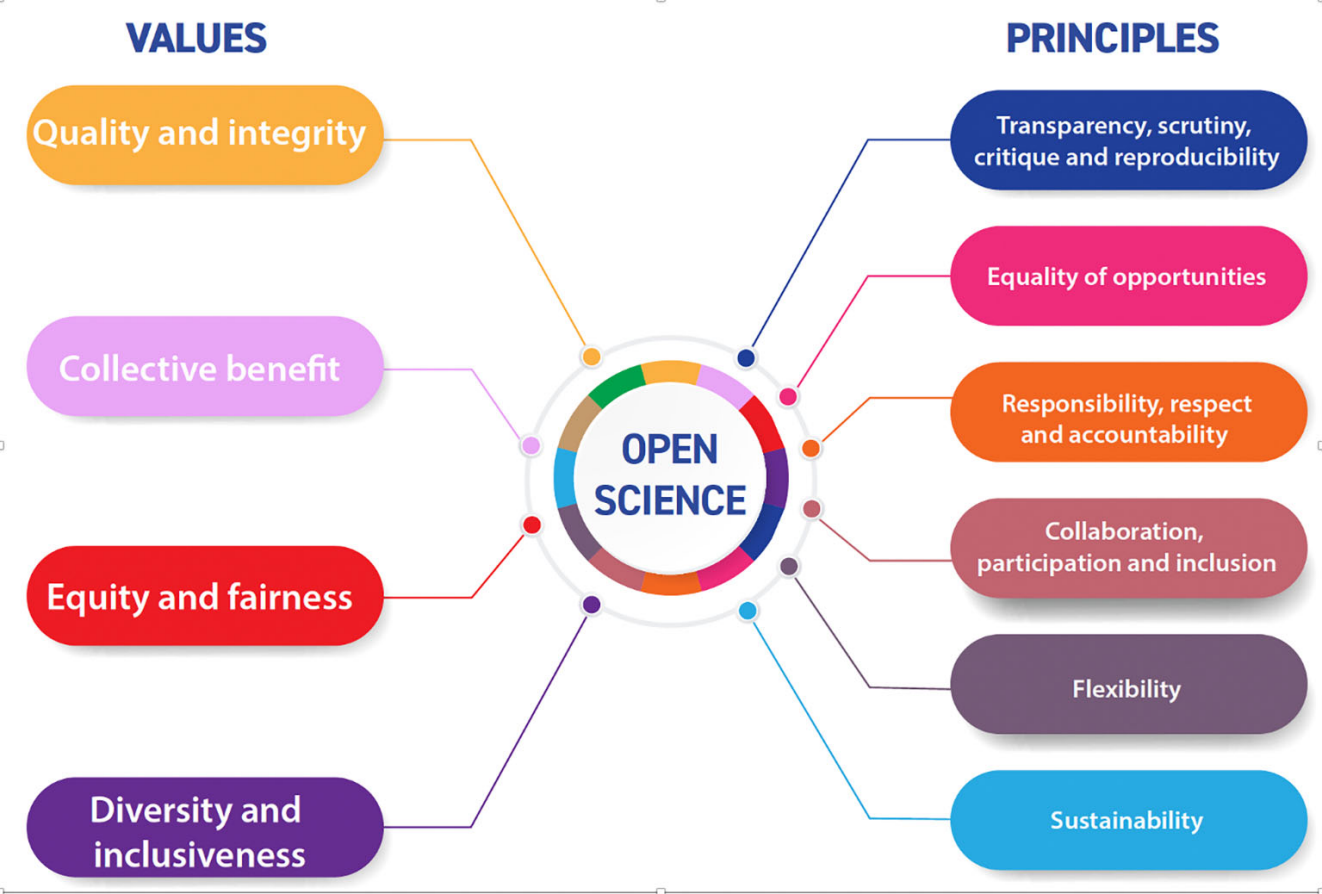
Values and principles mobilised by the UNESCO recommendation on Open Science. Source: UNESCO (2021).

The UNESCO recommendation on OS is the first internationally agreed definition of OS ( UNESCO, 2021). As a consensus document it exemplifies the breadth, ambiguity and transformative nature of OS discourses. This document made explicit two further steps. First, as illustrated in Figure 3, it highlighted the diversity of activities related to OS, particularly by giving more prominence to issues such as open engagement and dialogue with non-academic and marginalised actors (process-oriented practices), as already highlighted by the OECD ( Dai et al., 2018). Second, as shown in Figure 4, it made explicit the values and principles associated with OS. Some of these values and principles are related to rigor in scientific practices (quality, integrity, transparency, reproducibility, etc.). Others are more related to notions of justice within science (equity and fairness), and some more associated with the properties of the social contributions (diversity, inclusiveness, responsibility, participation) and their impacts (collective benefits). Many of these values are shared by other high level STI policy formulations, even by organisation’s which had traditionally focused on economic impacts, as illustrated in the OECD’s recent report ( OECD, 2023, pp. 87–121).

Both OECD and UNESCO’s comprehensive perspectives allow different stakeholders to highlight those activities and values which are most precious to them. However, this colourful palette also reveals that stakeholders hold a plurality of views on OS, with a battery of disparate activities. In consequence, its monitoring framework has to embrace this diversity of activities in a pluralistic way for its application in different contexts.

## 3. Open Science as a transformation of the research system

While stakeholders may not agree on the full range of activities or values of OS, a recurrent theme in OS discourse is that Open Science is about a *transition* or *transformation* of the research system ( Shelley-Egan et al., 2020). The transformation is seen as a more collaborative way of doing research, partly driven by the application in science of digitalisation and associated information and communication technologies ( Nielsen, 2011). However, it is also related to the perception of a major change in the contract between science and society, as discussed by various science policy discourses, including *Mode-2* and transdisciplinary research ( Gibbons, 1999; Nowotny et al., 2003), Responsible Research and Innovation (RRI) ( Owen et al., 2012; Shelley-Egan et al., 2020), mission-oriented research ( Mazzucato, 2018), Transformative Innovation Policies ( Loorbach & Wittmayer, 2024; Schot & Steinmueller, 2018) and science for SDGs ( Ciarli, 2022). In all these discourses, there is a call for transformative changes that are related to the changing relationship between science and society ( Shelley-Egan et al., 2020, p. 11).

Let us then take OS as transformation of the research system in all its dimensions: social, epistemic, institutional, organisational – even geographical ^9^. Then, OS would entail the evolution of the current research system towards a future research system in which free information flows and collaborative activities would play a more prominent role than they play nowadays. However, if we think of science as an evolutionary system with many-fold potential futures there is not one, but many potential OS futures, depending on the direction of change. This plurality of potential futures reflects the ambiguity of the concept and implies that the transformation it entails is open-ended, with different potential but uncertain futures ( Stirling, 2011).

Thus, the direction of this transformation will be given by the relative importance or the balance of efforts put into the different OS activities. A concentration of efforts around certain activities reflects a particular school of thought, and a particular model of what OS should be. From this evolutionary perspective, the key question is not if there is more or less OS, but which type of OS progresses, i.e. towards which type of (OS) practices the research system is being pushed and it evolves.

If one accepts that OS is a transformation of the science system, then the monitoring should aim at capturing the directions of transformative change. In the next section, we examine how changes in the models of science have been historically associated with reformulation of monitoring systems.

## 4. New models of science require new monitoring frameworks

Monitoring has the aim to check, show and justify that policy interventions, activities or organisations deliver the desired outcomes and impacts. Explicitly or not, monitoring and evaluation systems, as well as associated indicators, are derived from conceptual models on how some policies and activities lead to change towards the expected outcomes and impacts. These models guide the search of the properties that should be monitored and where possible measured ( Smith, 2004). If the models and systems under observation seriously evolve, the tools and methods of description also need to undergo a transformation. Yet monitoring tools and indicators are applied through existing (and thus old) institutional channels. As a result, efforts to capture new systemic changes are always difficult to adopt as they pose conceptual, technical, organisational and institutional challenges: institutions have a strong inertia to monitor according to past frameworks.

However, at some point monitoring and classification systems evolve in accordance with social transformations. For example, the logics of wine classification in bottle labels shifted from specific territory ( *terroir*) (as traditionally done in France) to wine grape varieties ( *cépage)* as a strategy by Californian companies in a context of globalisation in which the drinkers know little about local wine regions. Or dictionaries of commerce radically changed their classification of trade activities from the 18 ^th^ to the 19 ^th^ century, when the industrial revolution reconfigured the understandings of manufacture ( Douglas, 1986, pp. 91–109).

Let’s apply this idea of changes in monitoring to changing models of science. Schot and Steinmueller (2018) have proposed that there have been three main conceptual models about policies for science, technology and innovation (STI): the *linear model* (frame 1), the *innovation systems model* (frame 2), and more recently a model of *transformative research and innovation policies* (cf. Haddad & Bergek, 2023).

The linear (‘science-push’) model assumes an unproblematic flow from R&D investments to scientific knowledge, to technological inventions, and to market innovations that will eventually result in economic growth and increased well-being. Therefore, monitoring in policy frame 1 is conducted focusing on R&D inputs (investments) and S&T outputs. The OECD’s statistical manual for R&D data collection, known as the *Frascati Manual* ( OECD, 2015) is the main instrument to collect R&D investments. Scholarly publications are conventionally used as proxies for scientific output (the supply side), and patents as proxies for technological output ^10^. The linear model became increasingly questioned when it was realised that some countries (such as Japan in the 1980s) and organisations were very successful at innovation without being leaders in formal R&D. Publications and patents allow fine-grained analyses of the S&T contents, but they are poor in terms of tracing how and where this knowledge is used.

The perspective of the innovation systems model (frame 2) shifts the analysis towards the interactions between actors and the process of innovation in the firm ( Kline & Rosenberg, 1986; Smith, 2004). Here, the assumption is that innovation is best achieved when knowledge flows across academic organisations and industry. Since these processes of knowledge exchange were not captured in formal R&D measured by frame 1 statistics, the so-called *Community Innovation Surveys* (CIS) were developed to gather information directly from companies on how they create and introduce innovations. This effort consolidated in 1992 in the OECD’s *Oslo Manual* for innovation data in a long process that involved testing, trials-and-errors and learning through the engagement of policymakers and researchers ( Arundel & Smith, 2013; OECD & Eurostat, 2018). The *European Innovation Scoreboard* of the European Commission is another example of a monitoring framework based on the innovation system perspective. In this case, the approach takes a broad battery of about 25-30 indicators that describe different aspects of an innovation system, including education of human resources, attractiveness of research systems, R&D investments, innovation data (from CIS), firm and academic collaborations, and employment and sales impacts, among others.

We can take several lessons from these previous monitoring frameworks. First, their development takes time and is achieved through the creation of communities of practice at the interface of policy and research ( Godin, 2004). Second, monitoring frameworks are adjusted to adapt to evolving conceptualisations ^11^. Third, the focus of monitoring changes in accordance with the logics at the heart of each model: from inputs and outputs of formal research activities (frame 1) to the network of knowledge activities and introduction of market innovations (frame 2). In consequence of this drift in location, the new frameworks do not substitute but rather complement previous ones.

In recent decades, the idea that more innovation would always lead to more societal benefits has become increasingly questioned. It is now widely accepted that innovation in itself can lead to both desirable and undesirable societal impacts for example in terms of either lack of attention or negative impacts on health or the environment ( OECD, 2023, p. 87). As a response, a third model of innovation policy has been gradually emerging, with some STI policies explicitly directed at supporting transformative change towards desired goals in specific sectors such as food, health or mobility systems. This frame 3, known as Transformative Innovation Policies, shifts the focus towards innovation for transformative change in socio-technical systems such as those of energy, mobility or health, etc. particularly to address societal challenges such as climate change, urban sustainability, aging society ( Schot & Steinmueller, 2018; Weber & Rohracher, 2012). In frame 3, co-creation and transdisciplinary approaches are crucial to align research with societal needs.

Let us now take science as a socio-technical system in itself, the one concerned with the production of knowledge. If we then think of OS as a transformative agenda of this socio-technical system, as argued in the previous section, then OS policies can be understood as transformative policies for science. We can then apply the theoretical developments on Transformative Innovation Policies to OS. It might be argued that the research system differs from health, mobility or food systems in that it is not a tangible societal need. Still, the research system produces ‘knowledge’ for which there is a social demand, and then OS initiatives can be seen as innovations in the (socio-technical) system of knowledge production ( Loorbach & Wittmayer, 2024).

Let us then examine the recent insights on STI evaluation and monitoring from a frame 3 perspective, following the recent publication of a string of contributions ( Haddad & Bergek, 2023; Janssen et al., 2022; Kofler & Wieser, 2023; Molas-Gallart et al., 2021; Rohracher et al., 2023). In summary, we find that these studies (actually more focused on evaluation than on monitoring) suggest a shift in focus towards:

1. **A systemic perspective** of the various activities related to OS, including policies and outputs (e.g. software) but also processes (e.g. new evaluation methods in line with OS), outcomes (more inclusive recruitment of researchers) and expected impacts (e.g. scientific contributions to societal problems so far non-addressed);
2. **Learning** with regards to strategic choices and reflexive evaluation, with participatory processes and mixed methods if possible;
3. **Directionality of the activities and the values associated** with the choices in directions.

Since the purpose of monitoring in this frame 3 is not accountability or benchmarking, but learning (i.e. informing future strategy for specific different contexts), the methods and tools for conducting the monitoring do not need to be unidimensional indicators. Instead, new methodological tools can be created, for example showing collaborative networks, science maps or other visualisations that ‘open up’ reflexive thinking on the OS trajectories to be pursued ( Barre, 2010; Ràfols, 2019) ^12^.

In the next three sections we explore how these concepts could be applied in the context of a general OS monitoring, this is, a monitoring which aims to promote OS by capturing overall trends affected by a variety of (OS) policies, rather than assessing specific transformative programmes or policies.

## 5. Towards a systemic monitoring: broadening the palette towards processes and outcomes

Most initial initiatives towards monitoring OS have focused on describing whether research outputs such as publications, datasets or software are publicly accessible. Here we argue that a broader palette is needed for OS monitoring, for two reasons. First, because OS is defined as a broader set of activities than just outputs (which are the first pillar in Figure 3). Second, because public access to outputs in itself does not mean that the information or knowledge can be mobilised. As Michel Callon argued, science is not a conventional ‘public good’ because to make fruitful use of it, the potential beneficiaries need to invest or have invested previously in developing capabilities ( Callon, 1994). This is why firms invest in ‘absorptive capacity’, to ‘be able to recognize the value of new, external information, assimilate it and apply it’ ( Cohen & Levinthal, 1990, p. 128).

By focusing on accessibility without considering the capabilities needed to use information, there is the danger that outputs will not be utilized. Thus, policies have been developed to foster knowledge exchange, both within science (interdisciplinarity), and with specialised stakeholders (translational efforts, transdisciplinary projects and transfer offices) ( Molas-Gallart et al., 2016).

Since open outputs will mainly benefit those organisations or territories with absorptive capacities, generic open outputs from science may sometimes reinforce inequalities (see discussion on OD in the Global South ( Bezuidenhout & Chakauya, 2018; Bezuidenhout et al., 2017)). Development and innovation studies have documented that in many middle and low- income regions, science has not generated local benefits due to lack of local learning capabilities or absorptive capacity ( Arocena & Sutz, 2010; Bell & Pavitt, 1995). Yet, as Leonelli argues, genuine research openness is not about accessibility but about developing relevant interactions with the world ( Leonelli, 2023, p. 66).

An open publication (or dataset or software) may be formally accessible but created and communicated in such a way that it does not reach its potential beneficiaries. Either within or beyond academia, explicit efforts have to be made to search for users, to learn about their needs (participatory agenda setting), to engage them (co-creation), and to communicate to them the knowledge in meaningful ways (outreach). Therefore, a systemic perspective of OS also needs to track in what ways knowledge is created and used: whether it is generated and used through plural collaborations (processes) involving learning to and from stakeholder engagement (outcomes) and leading eventually to some social contributions (impacts).

Therefore, we propose that a systemic perspective would include a description of OS policies (enabling conditions) and OS outputs (e.g. publications), but crucially it should also include a monitoring of OS processes (e.g. public engagement, dialogue), outcomes (e.g. policy uptake) and, where possible, broader impacts (e.g. contributions to societal goals). This broadening of the monitoring to the full palette of scientific activities should allow to see changes towards more inclusive research processes, and whether the accessible scientific outputs are actually accessed and put to social use. Such a breadth is consistent with the ‘opening of monitoring’ and the ‘people-centred’ (process) framework suggested by the UNESCO OS Outlook ( UNESCO, 2023, pp. 27, 30). And this is important to avoid a streetlight effect ( Davies et al., 2021; Molas-Gallart & Rafols, 2018).

**Figure 5.**
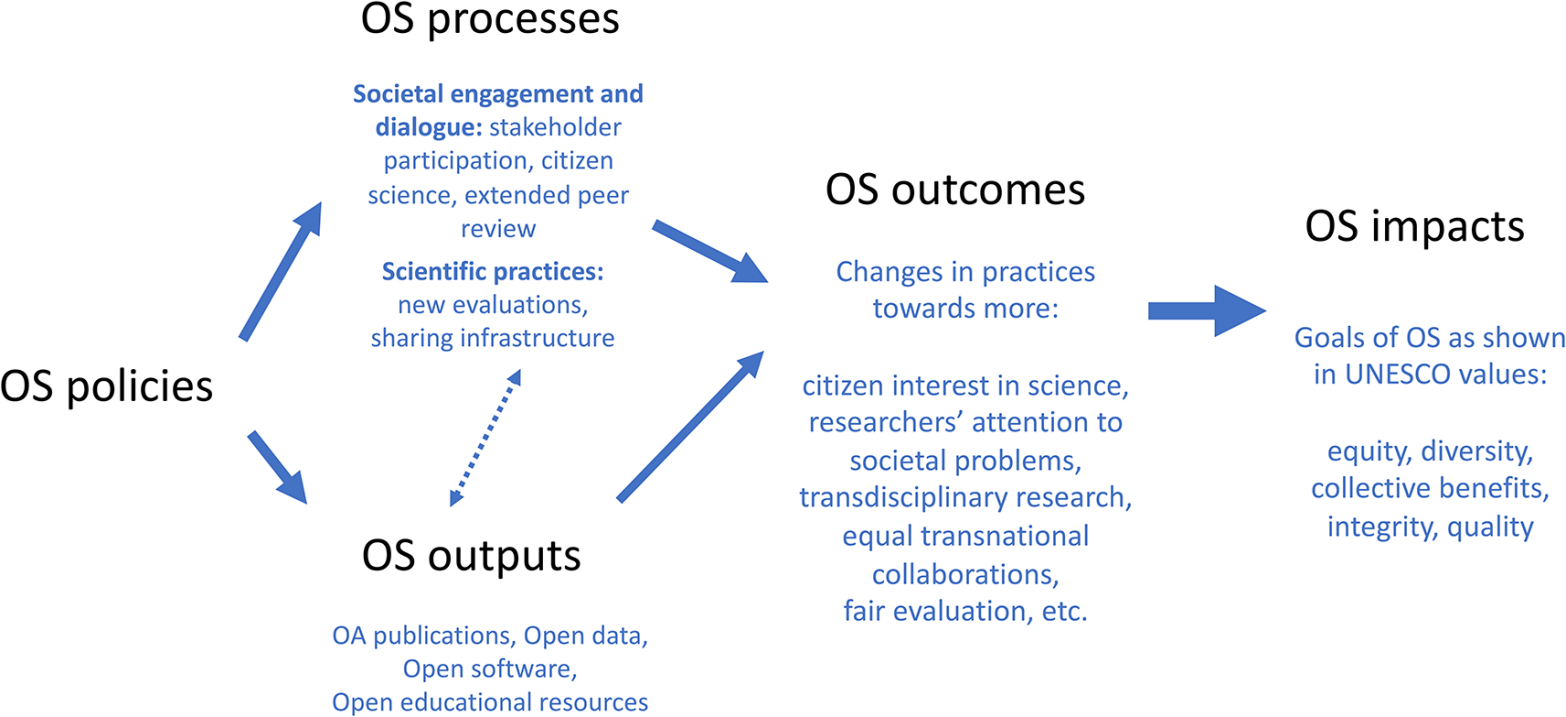
Different aspects of OS to be monitored: policies, outputs, processes, outcomes and impacts.

Figure 5 and Table 1 aim to summarise our proposal for a monitoring framework that is comprehensive and systemic, including the various aspects of OS: policies, outputs, processes, outcomes, and impacts. It should be noted that the distinction between processes, outcomes and impacts depends on the underlying evaluation theory, i.e. the specific theory of change on how the policy intervention achieves its goals. Since we are discussing OS policies in general, there is a degree of ambiguity regarding which actions can be considered processes, outcomes and/or impacts.

**Table 1.** Main aspects of OS monitoring, with examples of dimensions and monitoring efforts already conducted.

Aspect of OS	Examples of dimensions	Example of monitoring
**Policies**		
	… on OA publications	EOSC Observatory, national
	… on incentives rewards for OS	EOSC Observatory, national
	… on engagement	EOSC Observatory, national
	Institutional repositories	OpenDOAR, national
**Outputs**		
	Publications in OA	COKI Open Access Dashboard
	PhD theses	French OS Monitor
	Open datasets	DataCite
	Open software	French OS Monitor, Extracted from ORCID *
**Processes**		
	Open peer review	Graz U. survey ( Ross-Hellauer et al., 2017)
	Public engagement	SuperMoRRI, Bath survey ( Lawson et al., 2019)
	Sharing of research materials	Tokyo U. survey ( Shibayama et al., 2012)
	Integrity practices	Aarhus U. survey ( Schneider et al., 2023)
**Outcomes**		
	Policy utilization	Graz U. survey ( Cole et al., 2023)
	Fair evaluation in gender	See review in Sugimoto & Larivière (2023)
**Impacts**		
	Contributions of research to SDGs	Sussex U. analysis ( Ciarli, 2022; Purnell, 2022)
	Territorial distribution of research	Banaras Hindu U. ( Singh et al., 2021)
	Language diversity	COKI analysis
	Ethnic and gender distribution in the scientific workforce	U. Luxemburg ( Kozlowski et al., 2022)

* https://datacite.org/ see UNESCO Outlook ( UNESCO, 2023, p. 40).

For example, stakeholder engagement can be interpreted as a research practice (a process in itself), a change in a practice (and thus an outcome of the OS policy), and it may translate as goal in itself (an impact towards inclusion). We don’t think this ambiguity is problematic, as far as each dimension is transparently defined in a specific monitoring context. Thus, from a more nuanced perspective, the specific instances of monitoring can contextualise the aspect of stakeholder engagement captured, making the distinction between counting a given participatory event (a process), reporting of stakeholder learning during participation (an outcome), or reporting of inclusion of marginalised communities (an impact).

### 5.1 Current focus of monitoring: policies and outputs

The monitoring of **enabling conditions** refers to the description of dimensions that will facilitate or hinder the progress of OS. This comprises policies and institutional initiatives aimed at fostering OS, including specific OS action plans (e.g. infrastructure investment, training for FAIRisation of data) as well as transversal policies with major effects in OS (e.g. evaluation reform). The EOSC Observatory provides an example of policy monitoring at the EU level. The Finish OS monitor has designed a survey to institutions about their practices to support OS. The first step in UNESCO OS monitoring has been the development of a survey for the Member States to report their policies for promoting OS. ^13^ These surveys provide a solid ground for exploring institutional policies and commitments to OS.

The tracking of **OS outputs** in the form of OA publications or Open datasets is the most established monitoring practice. Bibliometric databases allow to conduct fine-grained analyses of the growth in the accessibility of the scientific literature. A problem is the uneven coverage of most commercial databases of across countries, disciplines and language, which marginalises knowledge relevant some world regions and communities ( Mongeon & Paul-Hus, 2016). Initiatives are under way across continents to address this issue ( Maricato et al., 2023). The French OS Monitor offers an example of the feasibility of rigorous analysis with highly comprehensive coverage using open scholarly infrastructure. Initiatives that make this tracking possible deserve to be valued and supported: for example, National CRIS systems like the Norwegian ( CRISTIN), regional bibliometric databases like SciELO (Latin America) and J-Stage (Japan), or portal for local OA scholarly journals (such as Catalonia’s raco.cat).

The tracking of open research data, open educational resources, open software, and open hardware is patchy but illustrative of growing efforts ( UNESCO, 2023, pp. 40–42), although the estimates of numbers of datasets, scripts or courses as general indicators is problematic given the large difference in size and efforts invested per unit.

### 5.2 Underdeveloped dimensions of monitoring: processes and outcomes

*Broadening out the palette of OS activities*

While counting outputs is a key monitoring tool, we should bear in mind that open outputs do not always lead to more OS. First, because, as argued above, since openness is situated and the capabilities of beneficiaries are unequal, potential access does not necessarily translate into the possibility of reaching, understanding and mobilising the knowledge ( Callon, 1994; Chan et al., 2020).

Also, because there can be instances in which the conventional ‘open’ routes of the outputs may not be associated with an increased flow of knowledge. For example, for a political scientist in Sweden publishing an opinion piece in Sweden in a newspaper behind paywall or in the local grey literature may achieve more readers and influence (in this sense more academic ‘openness’) than publishing an article in an OA academic journal in the US ( Salö et al., 2023). Or for an agricultural engineer in Colombia, publishing in Spanish in non-OA professional newsletter may be more important to reach her stakeholders (farmers) than publishing in English in a European OA academic journal ( Chavarro et al., 2017). For the particular case of policy influence of OS practices, Cole et al. (2023, p.1) study found that:

‘…there is little evidence that Open Research products, namely Open Access and open data (…) are useful in integrating science into policy-making. Instead, we found that the cognitive accessibility of research outputs is more important than their physical accessibility, and that inclusive and collaborative Open Research processes, like upstream engagement, co-creation and Citizen Science, are most effective at doing so.’

Processes of interactions such as collaborations and engagement are seen as separate dimensions to accessibility, and leading to different types of benefits ( Arza et al., 2017). This is why, in the UNESCO framing, as depicted in Figure 3, OS involves not only outputs (in green (1) pilar), but also research processes: in terms of collaboration in infrastructures (pink (2) pilar), stakeholder and citizen participation (orange (3) pilar), and dialogue with other knowledge systems (blue (4) pilar). These processes should also be monitored to assess progress in OS. Unfortunately, according to the recent UNESCO OS Outlook, they are poorly captured by OS reports at the moment: new methods need to be developed ( UNESCO, 2023).

Having made the argument that OS is a transformation of the research system, it is also important to monitor the changes in practices and behaviours induced by OS, i.e. the outcomes. Indeed, proposals for monitoring Transformative Innovation Policies agree that monitoring should focus on outcomes; that is, on assessing the ‘changes in the behaviour, relationships, activities, or actions of the people, groups, and organizations (…)’ ( Molas-Gallart et al., 2021, p. 435). Monitoring outcomes concerns questions such as how Open Data is re-used and by whom, and how public engagement influences research agendas.

One first approach to monitoring processes and outcomes is to approximate them through some relevant characteristics of the scientific outputs; for example, estimating frequency and type of exchanges with policy, industrial and civic organisations through formal co-authorship in publications ( Tijssen, 2006), or through the mention of scientific publications in social media or policy documents ( Díaz-Faes et al., 2019). But whereas many studies have looked at whether OA leads to more citations, let us emphasise here that for the benefits do not just lie on the amount of interaction and usage, but on the distributions across epistemic and geographical spaces. For example a recent study has reported that OA publications (even more if green OA) are cited in more diverse ways: they garner citations from more territories (countries and regions) and more disciplines ( Huang et al., 2024). This is a good example of an outcome (a change induced by OA: broader reach) aligned with the value of making science more diverse.

*The need to gather new data sources and surveys*

While analyses of usage of outputs are worth conducting and provide some information, qualitative studies have concluded that interactions between social actors and academics are extremely diverse ( Molas-Gallart et al., 2002). As a result, traces of interaction through some channels tend to be specific (biased) to certain socio-economic sectors and issues. For example, with some scientific issues (e.g. food consumption) are often mentioned in social media, while other (e.g. food production) receive more mentions in policy documents, and still others (e.g. agricultural methods) are only shared across professional information channels ( Noyons & Rafols, 2018). Moreover, many exchanges between academic knowledge and professions are not conducted through scientific publications, but through direct interactions and grey literature reports, often in the local languages ( Salö et al., 2023; Thune et al., 2023).

In conclusion, it is generally not possible to capture knowledge exchange trends reliably by using only traces left by conventional scientific outputs in digital platforms. In order to know how researchers share and use materials and data, how they interact with non-academics, etc., it is necessary to collect new data. Surveys are an obvious source of information. Some of the OS pilot monitors have started to conduct surveys. For example, the Finish monitor has proposed to conduct biannually a survey especially on ‘the development of services, policy documents, research assessment and culture of open scholarship’.

There is a long research experience of conducting surveys in order to understand the knowledge exchange practices in relation to a variety of collaborative practices ( Díaz-Faes et al., 2023), modes of knowledge exchange with industry ( D’Este & Patel, 2007), exchange with policy ( Thune et al., 2023), innovation practices in industry through the Oslo Manual ( Bloch, 2007; Gault, 2020; OECD & Eurostat, 2018), innovation in the public sector ( Arundel et al., 2019), and practices of Responsible Research and Innovation (RRI) ( Holtrop et al., 2022). Some studies aimed at monitoring OS processes also relied on surveys: for example on sharing of research materials ( Shibayama et al., 2012), open peer review ( Ross-Hellauer et al., 2017), policy use ( Cole et al., 2023), engagement with non-academics ( Lawson et al., 2019), integrity ( Schneider et al., 2023), or broader perceptions and habits of OS ( Ollé et al., 2023). To our knowledge, Arza et al. (2017) conducted the only survey looking into the multiple dimensions of OS, showing differences by field and region.

### 5.3 Monitoring potential impacts

Finally, there is the monitoring of the benefits of IS. It is common in evaluation theory to distinguish between ‘outcomes’ (changes in behaviours, practices, organisations directly induced by a policy intervention, e.g. more stakeholder participation), and ‘impact’ (referring to the ultimate changes that the intervention pursues such as societal benefits derived from scientific activity or gender balance) ( Molas-Gallart et al., 2021). Notice that in the UNESCO framing, the impacts refer the values expressed in Figure 4, which can be considered goals in themselves: more collective benefits, more equal, diverse and inclusive science. ^14^

We argue that the ways in which outputs, processes, outcomes and final impacts are related to each other should be mapped. In this way, monitoring may capture whether the evolution of the research system leads towards desired directions. This is important, because in a complex system one cannot assume that achieving the desired outputs (e.g. more open datasets) will lead to the desired outcomes (e.g. broader societal participation in specific research area) and desired impacts (the achievement of the collective benefits). Moreover, since the impacts are multifactorial, even if OS is pushing in a desired direction, other larger factors may counter the positive effects – and complementary policies may need to be considered. For example, evaluation reform may be needed to stimulate data sharing, because good open data repositories on their own may not be enough to trigger such behavioural change.

As potential dimensions of impact of OS, we propose those dimensions in the values of the UNESCO OS Recommendation, such as contributions to SDGs (collective benefits), distribution of research across countries (geographical diversity), distribution of research across languages (linguistic and cultural diversity), and balance in gender and ethnic group representation in scientific institutions (social inclusion).

## 6. Learning: reflexive monitoring for supporting strategic decision-making

Various studies have proposed that the learning function of evaluation should be emphasised in Transformative Innovation Policies ( Janssen et al., 2022; Molas-Gallart et al., 2021; Rohracher et al., 2023).

‘Reflexive monitoring and evaluation’ has emerged as a specific approach that distinguishes itself from more common ‘result-oriented’ evaluations by considering learning how to contribute to system innovation the central goal of evaluation. ‘Result-oriented approaches’ focus on accountability and steering, and on a set of predefined objectives, while ‘reflexive monitoring and evaluation’ put ‘the prevailing values and institutional settings up for discussion’ ( Van Mierlo et al., 2010, p. 36).

Kofler and Wieser (2023) have suggested the metaphor of ‘reflexive navigation system’ to convey the idea of monitoring for learning through engagement with stakeholders. The monitoring thus designed ‘organises information and knowledge flows, offering stakeholders an overview of achievements and challenges’, and this is used in expert panels in ‘regular exchanges and inclusive conversations with decision-makers’.

The idea is that the main use of monitoring should shift from accountability towards fostering thinking over strategic choices. In sociotechnical transformations there is great uncertainty regarding which of the many potential futures will be reached. This means, for example, that there is not one single trajectory or pathway to monitor, but a gamut of possible trajectories of which only one will be realised ( Stirling, 2011). Thus, in a transition towards a low-carbon energy technologies, a range of disparate energy portfolios is possible in a given territory or community (some dominated by wind-turbines, others by photovoltaics), with different environmental, security and economic consequences. Lessons learned from monitoring can facilitate debates towards re-balancing the portfolio in strategic ways, as exemplified in the RIPEET project.

Similarly, OS monitoring can facilitate this type of reflexive learning. For example, a given organisation has the potential to invest relatively more or less efforts across the activities showed in Figure 3, e.g. on how much resource goes to open data vs. engagement or collaborative infrastructure. OS monitoring of institutions can show that they have strengths and weakness over a portfolio of OS activities – and thus help reflection on developing strategies towards addressing these. The question is not whether the organisation has more or less OS (an accountability logic), but what types of OS it has and with what consequences in relation to its goals (a learning logic).

When monitoring aims to have a learning component, it should be comprehensive and contextual about the activities carried out (or not) (as discussed in the previous sections) and allow to reflect on the various available options. This type of information then empowers stakeholders to express preferences and make relevant normative choices. As put by the in the UNESCO OS Outlook, ‘[c] onsidering science as a global public good, it is important not to reduce open science to a few standardized metrics monitored in a top-down approach. Masking variations, particularly those between and within countries, may undermine the transition to a genuinely open science, accessible to all and with benefits for all’ ( UNESCO, 2023a, p. 18).

**Figure 6.**
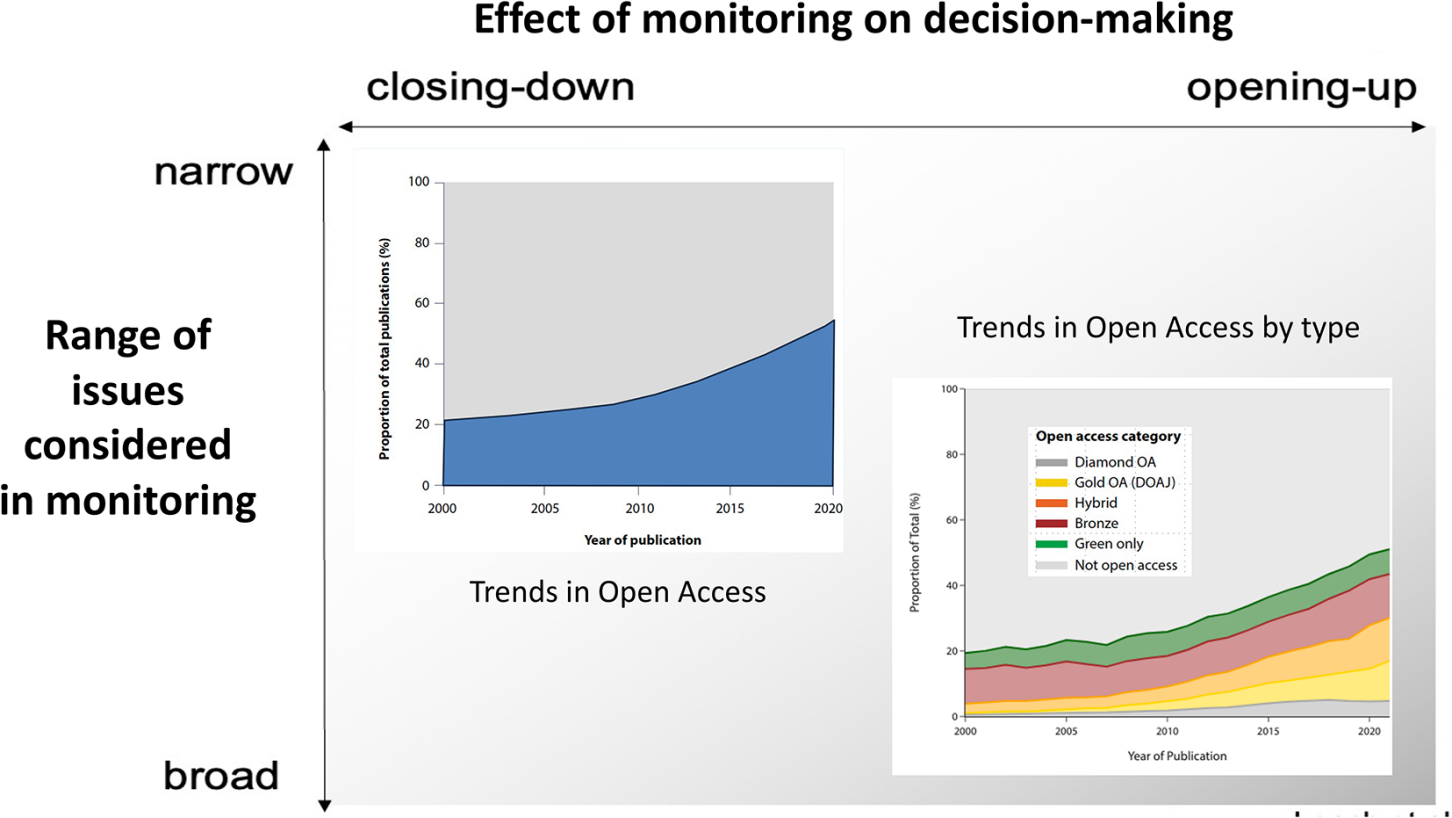
‘Broadening out’ (vertical) vs ‘opening up’ (horizontal) in monitoring for the case of Open Access. Legend: Framework of ‘broadening out’ (vertical axis: including more information in the analysis) and ‘opening up’ (horizontal: presenting an analysis allowing choices), based on Leach et al. (2010). Source of graph with trends: UNESCO (2023, p. 32).

Given the current policy inclination for narrow monitoring focused on accountability or efficiency, re-purposing monitoring to support learning will require new monitoring designs and training of users. This means in particular, applying monitoring methods in such a way that they facilitate the use of indicators as ‘debatable devices, enabling collective learning’ through contextualisation and stakeholder engagement ( Barre, 2010; Ràfols, 2019). To do this, monitoring can be enriched by ‘broadening out’ the range of issues considered in a given dimension (vertical axis in Figure 6) and by allowing users to ‘open up’ the indicators to scrutiny under various conditional perspectives (vertical axis in Figure 6) ( Leach et al., 2010; Stirling, 2008). Ideally, these ‘indicators frameworks’ would need to change according to different monitoring purposes and contexts ( Wouters et al., 2019).

For example, for Open Access monitoring an initial analysis might just focus into the growth of the number of OA publications (top left of Figure 6) and conclude that the trends are positive. By ‘broadening out’ the type of issues to type of OA, the analysis opens up a new perspective by visualising the types of OA which are growing fastest (gold and hybrid) – thus raising critical questions. A further step would be to consider the geographical distribution of publications. In contrast to rigid ranking lists which fostered narrow interpretations, the development in recent years of monitoring systems based on interactive visualisation platforms with filtering possibilities, has greatly facilitated ‘unpacking’ of indicators, mappings or collaborative networks ( Rafols & Stirling, 2021).

## 7. Directionality: visualising normative choices over trajectories of science

Directionality refers to the notion that science, technology and innovation (STI) systems follow certain trajectories (or pathways) rather than others. Evolutionary understandings have shown that STI systems develop along sociotechnical trajectories with path dependency ( Stirling, 2011). Different organisations or countries may follow alternative pathways. For example, Denmark committed to wind energy whilst France to nuclear energy as a result of divergent social and political commitments. Therefore, STI trajectories do not evolve over one dimension, but across a multidimensional space with a plurality of potential directions. There is a diversity of optional STI futures and different actors may favour different choices depending on their values and interests. A key insight from frame 3 of innovation policy ( Section 3) is that a specific STI system (e.g. mobility or energy) are locked-in in problematic pathways (e.g. due to pollution and CO _2_ emissions) and changes need to be supported towards alternative sustainable trajectories (e.g. wind-turbines, or nuclear).

‘Directionality means that innovation policy will not just stimulate specific technological options, but will look into the social and environmental drivers and consequences of each option, then aim for a deliberation on desirable pol-icy directions and eventually foster some desired directions for innovation, while blocking undesirable ones.’ ( Molas-Gallart et al. 2021)

To sum up, in any given transformation, the socio-technical trajectory is also associated with social commitments related to certain values. Monitoring should help in visualising alternative trajectories (wind vs. nuclear; green, diamond vs. gold OA). Where possible, the monitoring framework would inform of the broader social implications of each trajectory in terms of values, e.g. in alignment with SDGs, inclusion, distribution of benefits across stakeholders, etc.

In the case of OS, the notion of directionality means that OS will follow different trajectories, depending on the choices made regarding the adoption (or not) of particular commitments (e.g. FAIR, CARE datasets). A frame 3 perspective helps understand why monitoring the directions is so important: these choices define the OS future against alternative (not adopted) trajectories, often with broad, and sometimes unexpected, societal consequences.

Let us look for example, on how values are associated with different trajectories of Open Access in Figure 6. One of the key rationales for supporting an OA trajectory was to foster inclusion of researchers from Global South in transnational research communities through access to all journals. However, in the case of the gold and hybrid OA trajectories, access to reading is being (partly) achieved through the use of Article Processing Charges (APCs). Since these APCs are not affordable to many researchers in the Global South, these trajectories become a major barrier for researchers without resources to publish in highly visible journals or to publish in OA in hybrid journals, thus enhancing inequity.

In terms of outputs, these trajectories seem successful (they have higher % of Open Access articles), but the overall outcome goes against the value of inclusion. Olejniczak & Wilson (2020) show that men, older faculty, private and elite universities are more likely to publish in gold OA. This is a *Lampedusian* transition ^15^ in the sense that publishing practices change, but the incumbent actors (the large publishing corporations) preserve their interests and dominant position by using existing path dependency to shape the OS transformation towards those trajectories that favour them ( Larivière et al., 2015; Stirling, 2019). A monitoring strategy that is sensitive to value commitments, should not just look at the percentage of OA, but at the distribution of OA modes (green, gold, diamond, etc.) and its consequences in terms of equity, as illustrated in Figure 6.

Similarly, the apparently technical choices made on the implementation of Open Data (OD) can be analysed as representing different data sharing trajectories, with different ethical and social implications. On the one hand, data sharing is discussed according to the relative value of OD under different curation and infrastructure situations. This led to the proposal of the FAIR principles (Findable, Accessible, Interoperable and Reusable), with attributes that have implications in terms of the value of efficiency rather than fairness, in spite of its name ( Wilkinson et al., 2016). However, other framings like the *CARE Principles for Indigenous Data Governance* (Collective Benefit, Authority to Control, Responsibility, and Ethics) ( Carroll et al., 2020) put the values of equity and collective benefits at the centre, highlighting that there are major challenges on the usability of data by social actors ( Dosemagen & Williams, 2022), on data control and equity, and on the distribution of the benefits across Northern vs. Southern researchers ( Bezuidenhout et al., 2017) and stakeholders (e.g. communities endowed with genetic or natural resources). Given the implications of the different data sharing modes on OS values (as suggested by UNESCO), it seems relevant to include compliance or not with FAIR and CARE principles in a monitoring exercise. This means that, in terms of monitoring OD, the information should be provided with the particular OD trajectory (FAIR, CARE, both or other).

A third example is the choice regarding the governance data infrastructures in terms of centralised vs decentralised/federated architectures, which have implications on the geographical balance of power across countries and organisations, data control or accessibility ( Bezuidenhout & Havemann, 2020).

In summary, for each dimension of OS, there are alternative trajectories with different social implications.

## 8. Conclusions: ‘Opening up’ the monitoring of Open Science

As we are writing this paper in early 2024, many governments and scientific institutions have adopted OS policies and are developing methods to monitor progress toward the goals of OS. We observe that there is a tendency to focus the analysis on growth along those dimensions of OS that can be more easily measured, generally via the analysis of publications. However, these early measurements show already that ‘progress’ (adoption of OA) is not necessarily aligned with some of the OS policy goals (increased equity and inclusion in science) ( UNESCO, 2023).

Such choices have practical implications. Monitoring tools created by national governments and supranational organisations such as the UNESCO may appear to be far away from most OS research practices. However, one should not underestimate the influence of monitoring systems in shaping practices. Norms on how to develop and govern science are strongly influenced by (inter-)governmental institutions ( Finnemore, 1993) and practices on quantitative monitoring and evaluation constitute a key technology for governance ( Rottenburg & Merry, 2015). Traditional monitoring approaches have often suppressed diversity in research and its interactions with society in ways that run against the values of OS ( Rafols et al., 2012; Sugimoto & Larivière, 2023).

In this article, we propose an approach to monitoring that aims to engage with the complexity and ambiguity of OS ( Fecher & Friesike, 2014), and is aware of the potential suppression diversity associated with streetlight effects. Our point of departure is the assumption that OS is a transformation of the research system. Therefore its monitoring will require to map dimensions of research that have not been considered relevant in previous science models. Building on insights from the evaluation literature of transformative innovation policies, we have proposed strategies for monitoring OS (systemic view, learning and directionality), as summarised in Table 2 (compare with Leonelli (2023, p. 64, Table 3)).

**Table 2.** Summary of the shift proposed in monitoring strategies for Open Science.

	Mainstream OS monitoring	Proposed OS monitoring
**Evidence of OS transformation**	More open *outputs,* i.e. the products of research can be easily accessed	Reconfiguration of research system, in particular, changes in *processes and outcomes* likely to lead to improved social contributions
**Systemic change**	Few dimensions	Transformation in multiple dimensions
**Main OS concept**	OS as accessibility	OS as relevant connections
**Focus of analysis**	Science supply side (objects): publications, datasets, software, educational resources	Knowledge exchange processes and user/demand side (subjects): collaborations, engagement and dialogue events, usage of outputs
**Main purpose of monitoring**	Accountability and benchmarking	Learning and strategy
**Notion of directionality**	Absent	Directions as trajectories associated with normative preferences (i.e. choices in types of openness, e.g. green vs. gold OA), for each dimension
**Relationship to OS values and principles**	Absent or implicit	Making explicit the relations between monitored variables and desired impacts as related to collective benefits, diversity, inclusion, equity and integrity

First, we propose to ‘broaden out’ the dimensions to be monitored, adopting a **systemic** perspective which considers the various actions related to OS, including processes (e.g. participatory events), outcomes (e.g. use of knowledge in policy) and expected impacts (e.g. increased contribution to societal problems). This means going beyond the current focus on the science supply, on monitoring policies and outputs (e.g. datasets). Such a pluralistic perspective is warranted by an ongoing shift in the conceptualisation of OS from ‘accessibility to research outputs’ towards ‘judicious interactions’ ( Leonelli, 2023) both in the scientific practices and the societal usages of research. The type of information needed cannot be garnered only from research output data in digital platforms. Therefore existing data sources on outputs (objects) will have to be complemented with new data sources on researchers’ OS activities, especially interactive processes. Given that many collaborative practices and science-society exchanges are invisible in outputs or digital platforms, we believe that direct inquiries are needed, via surveys or qualitative approaches, to understand OS dynamics.

Second, given the uncertainty associated with sociotechnical transformations, and the unexpected outcomes of transformative policies (as illustrated by increased publishing inequity generated by OA), monitoring should be oriented towards supporting reflective **learning** – rather than a focus on accountability and benchmarking. Monitoring should be understood as a navigation instrument that helps to set sails towards the desired goals in the face of hidden currents and shifting winds.

Third, monitoring should be ‘opened up’ by making **directionality** visible. Since there a many potential futures in a transformation and contending visions, monitoring OS should not be only about tracking more or less OS, but about what trajectories of OS are developed and with what consequences. Different trajectories (e.g. green vs. gold OA; FAIR vs. CARE OD) are associated with different interests and values – and therefore lead to different OS futures. In order to map choices, it is necessary to adopt multidimensional monitoring tools and visualisations.

A final consideration is the diversity of contexts of science and society. This means that flexibility (an OS principle in the UNESCO Recommendation) will be required to apply monitoring in different locations or organisations ( Wouters et al., 2019). The strength of a monitoring framework is its capacity to summarise a diverse world. But this can become a liability without a good dose of humility and reflexivity ( Stirling, 2023, p. 5). As Leslie Chan reminds us:

‘… there is no single or universal concept of Open Science that is sufficient to encompass the diversity of knowledge traditions and practices from around the world. Hence the term Open Science and the notion of “openness” is highly situated, constantly subjected to negotiation according to local contexts and historical contingencies. Our collective observations therefore challenge the tendency to define Open Science as a set of technical infrastructure, workflow, protocols, and licensing conditions that can be universally applied regardless of context, history, and human agency.’ ( Chan et al., 2020, p. 17)

Just like the wealth of ecosystems in the Earth cannot be described by simply counting the number of individuals of each species, the transformation towards OS cannot be described by counting open outputs. Instead, the evolution of ecosystems is understood through the changes in the network of interactions among species in each ecosystem. Similarly, the evolution of OS needs to be monitored through the trajectories in the exchanges of scientists and citizens – learning and adapting dynamically in situated knowledge ecosystems.

## Data Availability

No data are associated with this article.

## Footnotes

^1^

https://research-and-innovation.ec.europa.eu/strategy/strategy-2020-2024/our-digital-future/open-science/open-science-monitor_en

^2^

https://frenchopensciencemonitor.esr.gouv.fr/software/general

^3^

https://avointiede.fi/en/policies-materials/monitoring

^4^

https://eoscobservatory.eosc-portal.eu/home

^5^

https://unesdoc.unesco.org/ark:/48223/pf0000383805

^6^
It should be noted that the breadth of the concept of science is ambiguous. In accordance with most science policy, here we take a broad definition: we use it for all scholarly disciplines including social science and humanities, as well as research activities outside of academia. We would prefer using the terms *research* and *open research*, but we keep the term *science* in order to align with current policy discourse on OS.

^7^
While their framing in terms the five schools is possibly outdated, it illustrates that very disparate understandings on OS have long existed.

^8^
The recent addition of ‘trust in science’ shows again the political nature of the definition of OS, now a major issue following the battle for public confidence in COVID-19 responses.

^9^
A more inclusive science should be more less concentrated in rich nations and rich cities ( Ciarli, 2022; UNESCO, 2023, p. 36).

^10^
See for example, the description of ‘U.S. and Global Science and Technology Capabilities’ in the Science and Engineering 2022 report.

^11^
The Oslo manual (as the Frascati) has undergone various revisions. For example the definition of innovation has broadened from only technological product and process innovation in the first edition (1992), to include also non-technological products and processes, new marketing methods, or a new organization method in business practices, workplace organization or external relations, in the third edition in 2005. Also, an annex was added to the Oslo Manual in 2005 for its use in developing countries, after the experience provided by the Ibero-American Network of Indicators of S&T (RICYT) in *Bogotá Manual* ( Gault, 2013, p. 51). In recent years, proposal have been made to include public sector innovations (which are not counted currently since they are not market-oriented), social and household innovations and innovations in the informal sector ( Gault, 2020). Similarly, the battery of indicators of the Innovation Scoreboard has evolved over time – for example, with the introduction of indicators of environmental sustainability in recent years (which is a hint of a shift towards frame 3).

^12^
We thank Anestis Amanatidis for suggesting this point.

^13^

https://unesdoc.unesco.org/ark:/48223/pf0000386862

^14^
Here it should be noted that, if inclusiveness or integrity are considered to be instrumental properties (rather than a value in itself ), then they will be considered a change in practice (an outcome) that is expected to contribute to higher order values (for instance by contributing to the achievement of social goals).

^15^
After Tommaso Di Lampedusa, the author of the novel *The Leopard* which describes how the old Sicilian aristocracy preserved their power through the unification of Italy and the ascent of capitalism: ‘Everything must change for everything to remain the same’.
