# Supplementary material for: Monitoring Open Science as transformative change: Towards a systemic framework
Source: F1000Res. 2024 Apr 23;13:320. [Version 1] doi: 10.12688/f1000research.148290.1 (PMC11157193; doi:10.12688/f1000research.148290.1)
Supplement: Peer review history

# Reviewer report: Reviewer response for version 1

**DOI:** 10.5256/f1000research.162580.r282405

**Published:** 2024-06-06

**Authors:** Francesca Di Donato

**Affiliations:** 1 Consiglio Nazionale delle Ricerche (CNR-ILC), Pisa, Italy

The article first discusses the plural understandings of Open Science (OS), focusing on the OS definition provided by the UNESCO Recommendation of 2021, endorsed by 193 countries, which provides the most complex yet inclusive definition available. This definition implies a shift from an understanding of OS as the ‘possibility of access’ towards an understanding as the ‘process of connection’ in accordance with values related to integrity and epistemic justice.

 Based on this premise, the article makes the case that OS is a systemic transformation of research in all its dimensions (social, epistemic, institutional, organisational  and geographical). It then reviews how models of science are associated with particular monitoring frameworks, considering several lessons learnt from the existing ones and how they complement each other, and it specifically focuses on the frame known as Transformative Innovation Policy (TIP), which shifts the focus towards innovation for transformative change in socio-technical systems such as those of energy, mobility or health, particularly to address societal challenges. 

 Furthermore, the paper describes concepts that can be useful for monitoring OS as a transformation, suggesting a shift in monitoring frameworks for OS towards three directions, aimed at adopting: (i) a systemic perspective, switching from an outputs oriented perspective towards a wider one, focusing on policies and outputs, processes, outcomes and impacts; (ii)  a learning approach to monitoring, i.e. helping taking decision considering diverse options within OS; (ii) and a directional perspective, which considers the different trajectories within OS dimensions and the associated values and potential effects. 

 The opinion article is based on a very accurate analysis of the current literature, including a documented literature analysis which supports the developed arguments; in particular, all factual statements are adequately supported by citations, and the conclusions are on these basis fully justified.

Is the topic of the opinion article discussed accurately in the context of the current literature?

Yes

Are arguments sufficiently supported by evidence from the published literature?

Yes

Are all factual statements correct and adequately supported by citations?

Yes

Are the conclusions drawn balanced and justified on the basis of the presented arguments?

Yes

Reviewer Expertise:

Political philosophy, Open science, Research on research

I confirm that I have read this submission and believe that I have an appropriate level of expertise to confirm that it is of an acceptable scientific standard.

# Reviewer report: Reviewer response for version 1

**DOI:** 10.5256/f1000research.162580.r282406

**Published:** 2024-06-05

**Authors:** Hans De Jonge

**Affiliations:** 1 Dutch Research Council, The Hague, The Netherlands

In this opinion article the authors argue that the way open science is being monitored is in need of a fundamental revision. Traditional open science monitors have very much focused on outputs using quantitative approaches: analyzing the number of open access publications over time. More recently, these reports have been complemented with attempts to monitor open availability of research data and code / software. The authors – in my view, rightly – argue that these narrow approaches can lead to streetlight effects (counting what can be counted) and misrepresentation of the much broader open science agenda as systematic transformation of the research system. Informed by the literature about Transformative Innovation Policies the authors call for a shift along three lines:

  

- The dimension of monitoring should be broadened as to include not only (more) outputs but also processes, outcomes and impacts.
- Open Science monitoring exercises should be more directed towards learning instead of only accountability and benchmarking.
- Monitoring should open up by making visible the ‘directionality’, especially the problematic sometimes unintended consequences of certain choices.  

Given the enormous proliferation OS monitoring worldwide I think the authors view is very valuable and topical. The article is well structured and overall, well written (see editorial notes below) and is very well supported by the most recent literature on the topic.

 I have only a few comments and leave it to the authors to take them into consideration or discard them.

  

- **Directionality. **

I struggled with the concept of directionality. It is only very briefly introduced in the abstract and the introduction (p. 4) and again on p. 10. but for me not to an extend that I understood what was meant by it. I didn’t find the examples (“e.g. different colour of the routes to OS”) very helpful. Only on p. 16 the authors explain that directionality means that in the adoption of certain OS practices different choices (trajectories) can be made, that some of these trajectories can have unintended consequences or even outcomes that are outright in conflict with the values of open science (three examples are given that conflict with the equity value). I completely concur with the authors that being open to negative, unintended or conflicting consequences need to part of a more comprehensive open science monitoring but I think some readers might need some help in understanding this point throughout the article.

  

- **Schools of thought**

I am very happy to see that the authors take the effort of making clear that Open Science is a very broad concept which encompasses a multitude of activities, practices and agendas. In that context reference is being made to the seminal work of Fecher and Friesike who have argued that under the broader umbrella term of Open Science five schools of thought can be distinguished. Each school has its own focus, depending on what each school thinks is the most pressing problem in the current science system. While I applaud the authors for emphasizing the plurality of the Open Science movement, I cannot completely escape the impression that they themselves feel more closely associated with one particular school (the public school) and less with others, leading to – in my view - a somewhat narrow definition of what Open Science is aiming for. Two examples to make this clear.

  

1. First, when arguing that monitoring should not only focus on policies and outputs but also on processes and outcomes, this is consistently illustrated by the authors with examples from the intended ‘societal’ benefits of OS: OS leading to more citizen engagement, researchers more focusing on societal problems, more participatory events, open engagement and dialogue with non-academic actors. (Also in figure 5 and table 1 outcomes and impacts of OS are only defined in terms of societal benefits). While I agree that these are very important objectives of OS, I do not think they are the only ones. Much of what is being done in Open Science is – I would argue – to improve to research process itself. To make science more transparent, more reproducible, more efficient, more interoperable, the dissemination more quick and efficient. All to ensure the quality of science increases and trust in science (by scientists and society) increases. This notion that one of the intended and hoped for outcomes of Open Science is increased quality and integrity is markedly missing from the narrative of the authors, although quality and integrity is the first of the 4 values mentioned by UNESCO and the authors stress the importance to focus monitoring OS on these UNESCO values.
2. A dimension that is – in my view - also somewhat under represented is that one of the ‘collective benefits’ of Open Science is its potential economic impact. Although I agree, that many of the initial aspirations (for instance the Budapest Declaration) were to do with global equity and fair participation of underrepresented parts of the world in the research system, one cannot deny, that a lot of the policy debates around open science are motivated by considerations around unlocking publicly funded research for innovation, knowledge transfer and economic growth. Indeed, much of the early government support for Open Access in countries like the NLs and the UK were motivated by Open Science as potential driver for economic impact. Apart from the quality/trust motivation, this economic dimension is also absent from the authors narrative.

I would encourage the authors to think of their own position in the ‘five schools of thought’ and consider whether in presenting examples of how to monitor OS outcomes and OS impacts they have struck the right balance. Do these examples cover the broadness of the Open Science agenda and the plurality of activities, practices and motivations that are currently covered by Open Science?

  

- **UNESCO survey**

The work of this opinion article is being done as the result of the authors’ engagement with the UNESCO working group on Open Science monitoring. The authors present a very interesting framework for future Open Science monitoring which is much more comprehensive and systematic in nature than much of the older monitoring exercises. For the moment, however, it seems that UNESCO has not chosen to adopt this model, which – to me at least – feels like a missed opportunity. Instead, UNESCO will put out a survey which – at this stage at least – will focus on supporting actions taken by member states to foster open science. I would be interested to learn the authors view on this decision and since this is an opinion article encourage to take a more critical stance, maybe as part of footnote 13.

**Minor issue:**

 Since the writing of this article an important initiative has been launched to support the move towards open bibliometric metadata: the Barcelona Declaration. This is at least partly motivated by the increase in open science monitoring activities and the importance to base these monitors on open instead of proprietary data. The authors might consider referring to this initiave as part of paragraph 5.1 (p.12).

**Editorial:**

 Change ‘gathering statistics’ to ‘collecting statistics’ p. 3

 Change ‘this study is prompted’ by ‘this article is prompted’ p. 3

 Change ‘for addressing of societal problems’ to ‘for addressing societal problems’

 Change ‘the one concerned with’ to ‘one concerned with’ p. 9

 Change ‘FAIRisation’ to ‘FAIRification’ p.12

 Change ‘most commercial databased of across countries’ to ‘most commercial databased across countries’

 Elaborate this statement: ‘The tracking of open research data, open educational resources, open software, and open hardware is patchy but illustrative of growing efforts (UNESCO, 2023, pp. 40–42), although the estimates of numbers of datasets, scripts or courses as general indicators is problematic given the large difference in size and efforts invested per unit’. I don’t understand the problem.

 Table 1 p. 11 Change ‘Extracted from ORCID’ – I am not sure what is meant. The French OS monitor can be found online.

 Tabel 1 p. 11 ‘Integrity practices’ – consider ‘Open methods’ as an umbrella term for all methods like preregistration, open reporting, registered reports, etc.

 Change ‘(in the sense more’ to ‘(in the sense of more’ p. 13

 Change ‘publishing in Spanish in non-OA professional newsletter’ to ‘publishing in Spanish in a non-OA professional newsletter’ p. 13

 Review this sentence: ‘let us emphasize here that for the benefits do not just lie on the amount of interaction and usage, but on the distributions across epistemic and geographical spaces’ p. 13.    > ‘for’ seems to be not right here?

 Rewrite ‘even more if green OA’ > too informal?

 Change ‘IS’  to ‘ OS’ p. 14

 Change ‘impacts refer the values’ to ‘impacts refer to the values’ p. 14

 Consider to further support the suggestion: ‘As potential dimensions of impact of OS, we propose those dimensions in the values of the UNESCO OS Recommendation,’  p. 14

 Review quotation ‘to contribute to system innovation the central goal’ p. 14 > word seems missing. Innovation ‘as’?

 Change ‘As put by the in the UNESCO OS Outlook’ to ‘ As put in the UNESCO OS Outlook’ or even ‘As argued by UNESCO OS Outlook’ p.15

 Change ‘By ‘broadening out’ the type of issues to type of OA’ to ‘By ‘broadening out’ to type of OA’ p. 15

 Change ‘pol-icy’ to ‘policy’ p.16

 Explain ‘A frame 3 perspective’ p.16

Is the topic of the opinion article discussed accurately in the context of the current literature?

Yes

Are arguments sufficiently supported by evidence from the published literature?

Yes

Are all factual statements correct and adequately supported by citations?

Yes

Are the conclusions drawn balanced and justified on the basis of the presented arguments?

Yes

Reviewer Expertise:

Open Science, monitoring

I confirm that I have read this submission and believe that I have an appropriate level of expertise to confirm that it is of an acceptable scientific standard.
